# A novel *CUL7* mutation in a Japanese patient with 3M syndrome

**DOI:** 10.1038/s41439-018-0029-3

**Published:** 2018-10-23

**Authors:** Tomozumi Takatani, Tadashi Shiohama, Rieko Takatani, Naoki Shimojo

**Affiliations:** Department of Pediatrics, Graduate School of Medicine, Chiba University, Chiba, Japan

## Abstract

3M syndrome is an autosomal recessive disease characterized by severe pre-natal and post-natal growth retardation, dysmorphic facial features, and skeletal abnormalities. We present a patient with 3M syndrome caused by the compound heterozygous mutations p.Trp68* and p.Gly1452Asp in *CUL7*, the latter of which is novel, who exhibited a good body height response to growth hormone treatment. These results expand our knowledge of phenotype–genotype correlations in 3M syndrome, including correlations relevant to growth hormone response.

3M syndrome (MIM #273750) is an autosomal recessive disease characterized by severe intrauterine and postnatal growth retardation with dysmorphic facial features and skeletal abnormalities^1^. Affected individuals have a triangle-shaped face, a pointed chin, frontal bossing, prominent ears, an upturned nose with a fleshy tip, a long philtrum, and full, fleshy lips. They can also have a short neck, square shoulders, winged scapulae, a short thorax, a chest groove, pectus carinatum or excavatum, hyperlordosis, scoliosis, joint hypermobility, clinodactyly, spina bifida occulta, hip dysplasia, and/or prominent heels. However, these features are variable and nonspecific. It can be difficult to distinguish 3M syndrome from other diseases or syndromes involving dwarfism; therefore, genetic testing is valuable.

Three different genes, *CUL7* (MIM #273750)^2^, *OBSL1* (MIM #612921)^3^, and *CCDC8* (MIM #614205)^4^, have been identified as being responsible for 3M syndrome, with *CUL7*, *OBSL1*, and *CCDC8* mutations found in 65, 30, and 5% of 3M syndrome patients, respectively^5^. The growth hormone (GH) response varies widely among individuals with 3M syndrome. Certain studies have found GH treatment to be beneficial, whereas other investigations have described such treatment as having no effect. In part, the response of individual patients to GH depends on their specific causative genes for 3M syndrome. For instance, GH treatment produced a better response in a patient with a *CCDC8* mutation than in a patient with an *OBSLB1* mutation^6^. To date, a good response to GH treatment has been reported for only one patient with a *CUL7* mutation^7^. Due to the rarity of 3M syndrome, insufficient data are available for phenotype-genotype correlation studies, particularly research that addresses response to GH treatment. The patient described in the present study expands our understanding of phenotype–genotype correlations in 3M syndrome, especially with respect to the GH response.

The patient is a 7-year-old boy with nonconsanguineous parents. He was born by vacuum extraction at 41 weeks gestation without asphyxia. His birth weight, length, and head were 2246 g (−3.47 SD), 47 cm (−1.85 SD), and 33.5 cm (−0.34 SD), respectively. He exhibited hypocalcemia 7 days after delivery because of transient neonatal hypoparathyroidism. The administration of alfacalcidol and calcium lactate normalized his calcium levels. A follow-up examination after 3 years revealed normal cognitive development and no catch-up growth. The patient showed proportional growth and severe short stature as well as frontal bossing and a triangular face. Radiographic examinations showed slender long tubular bones and delayed bone age. A blood test revealed normal insulin-like growth factor-1 (IGF-1) levels. In accordance with guidelines for GH treatment for short children born small for gestational age^8^, GH therapy for short stature was started at 3 years of age; at this time, the patient’s height was 78.2 cm (−4.64 SD). His growth velocity was 7.1, 7.0, and 6.5 cm/year in the first, second, and third years of GH therapy (Fig. 1). GH was started at 0.23 mg/kg/week; currently, at 7 years of age, the patient receives GH at a dose of 0.45 mg/kg/week. When he was 5 years of age, an insulin GH stimulation test showed normal GH secretion.

**Fig. 1:**
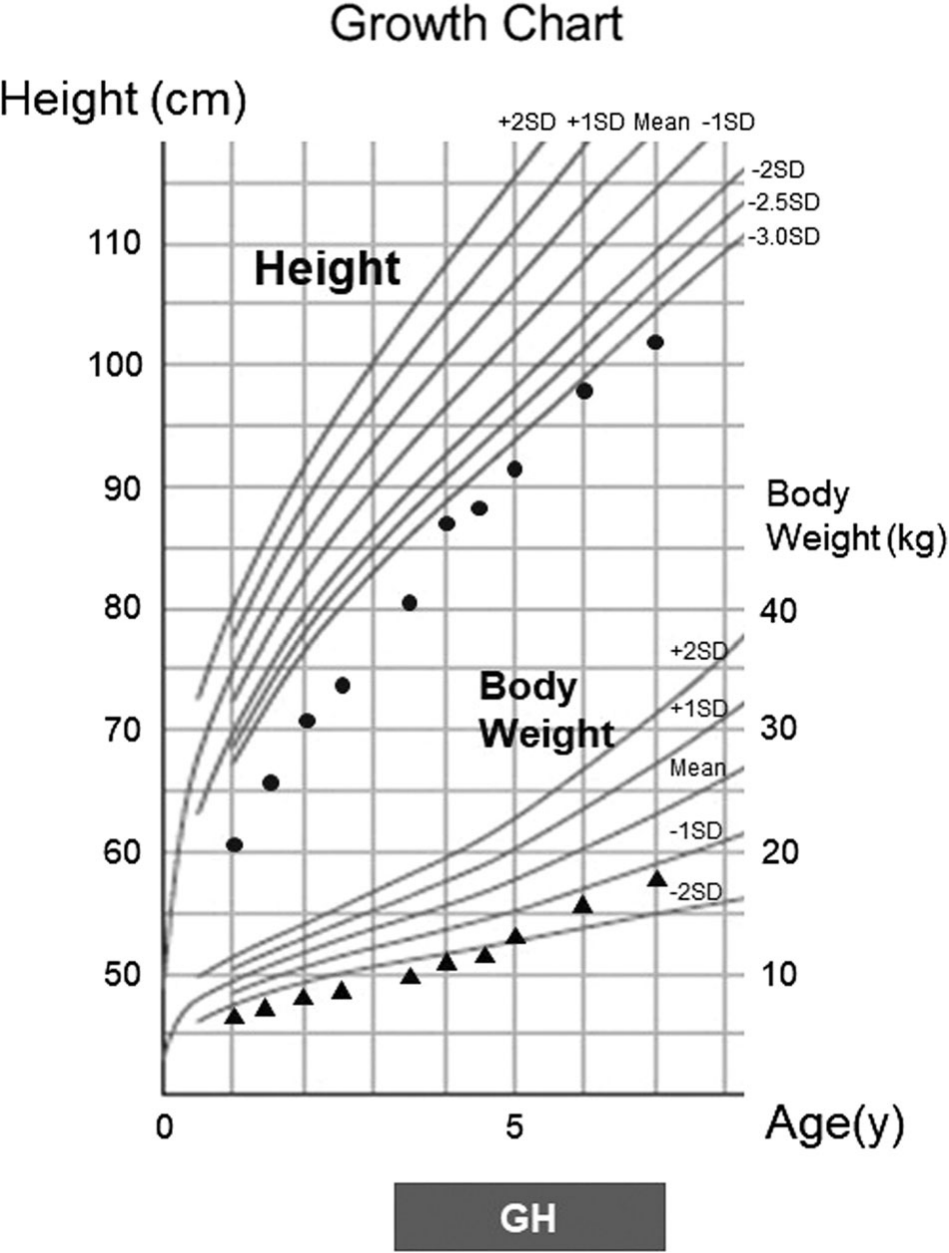
Height and body weight growth chart for the patient

Genetic analysis approved by the Institutional Review Board of Chiba University was planned to determine the cause of the patient’s short stature. After written informed consent had been obtained, genomic DNA was extracted from peripheral blood samples from the patient and his parents. Genetic analysis was performed using an Illumina TruSight One sequencing panel on a MiSeq platform in accordance with the manufacturer’s instructions (Illumina, San Diego, CA, USA). Sequencing reads were analyzed using Basespace VariantStudio ver. 2.2 (Illumina). Mutations were validated by standard Sanger sequencing. We identified the compound heterozygous mutations NM_014780.4 (CUL7_v001): c.203G>A [p.Trp68*] and NM_014780.4 (CUL7_v001): c.4355G>A [p.Gly1452Asp], which were carried by both parents (Fig. 2a). The latter is a novel mutation. The missense mutation p.Gly1452Asp was predicted to be deleterious by SIFT^9^ and probably damaging by PolyPhen^10^. Multiple alignment of CUL7 amino-acid sequences using Clustal Omega^11^ showed that glycine at position 1452 is highly conserved across species (Fig. 2b).

**Fig. 2:**
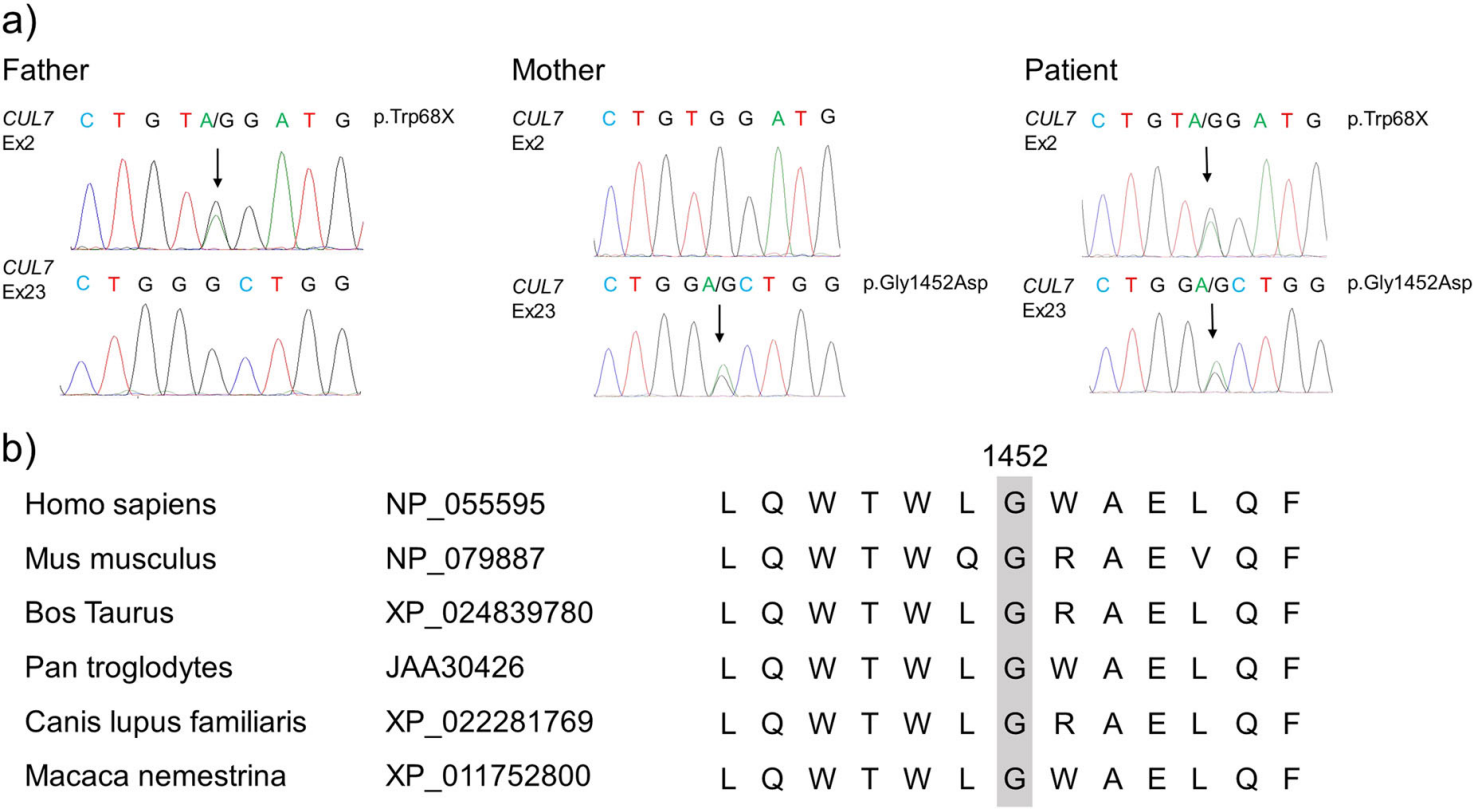
**a** DNA sequence chromatograms showing the heterozygous mutations c.203G>A (pTrp68*) in the father and c.4355G>A (p.Gly1452Asp) in the mother. Both mutations are detected in the patient. **b** Multiple sequence alignment of CUL7 amino-acid sequences from different species performed using Clustal Omega (PMID: 25501942). The RefSeq (https://www.ncbi.nlm.nih.gov/refseq/) protein identification of CUL7 for each species is shown.

Here, we describe a boy affected by 3M syndrome caused by mutations in *CUL7* and report his growth before and after GH administration. To date, only one report has shown that GH administration improved the height of a patient with 3M syndrome caused by compound heterozygous mutations in *CUL7*; in that study, the patient’s growth velocity was 8 cm/year during the first year of GH therapy, which was started at 7 years of age, and 6.5 cm/year during the second and third years of GH treatment^7^. Our patient showed a similar improvement in growth. A higher GH dose is currently administered to our patient (0.064 mg/kg/day) than was given to the previously reported patient (0.045 mg/kg/day). Another report of 3M syndrome caused by a homozygous 10 bp deletion in exon 18 of *CUL7* detailed a poor response to GH treatment started at 1 year of age^12^. That patient also showed a GH deficiency. Differences in response to GH may be attributable to GH dose or the specific mutations involved in various cases.

CUL7 was previously described as the ubiquitin ligase for insulin receptor substrate-1 (IRS1), which is essential for GH–IGF1 signaling. CUL7 deficiency leads to increased IRS1 protein levels in vitro. Therefore, it was thought that IGF-1 signaling, via IRS1, was decreased in 3M syndrome. However, previous results were obtained by investigating the effects observed when CUL7 was almost completely absent. Our patient has, at least from one allele, the full CUL7 sequence, which could account for the observed differences in the IGF-1 response between our patient and prior patients. However, more detailed data regarding genotype–phenotype correlations is required to reveal the effectiveness of GH treatment in 3M syndrome. Additionally, because *CCDC8* and *OBSLB1* are also causal genes of 3M syndrome, another mechanism could underlie the dwarfism caused by mutations in these genes because their encoded proteins function independently of IRS1. Indeed, several reports have shown that the 3M complex regulates chromosome integrity^13,14^. Moreover, mutations in the 3M complex alter microtubule dynamics, leading to reduced cell proliferation^13,14^. These facts also support the need for further study of 3M syndrome patients.

Targeted next-generation sequencing can provide a definitive molecular diagnosis of 3M syndrome that suffices for both determining disease prognosis and providing appropriate genetic counseling. Indeed, several reports have highlighted the usefulness of next-generation sequencing for diagnosing 3M syndrome patients, many of whom might have remained undiagnosed without this approach^15–17^. Our patient further supports the effectiveness of the clinical application of next-generation sequencing for 3M syndrome. The increased use of next-generation sequencing for 3M syndrome candidates could also expand our knowledge of genotype–phenotype correlations, including correlations relevant to response to GH treatment.

## Data Availability

The relevant data from this Data Report are hosted at the Human Genome Variation Database at 10.6084/m9.figshare.hgv.2372; 10.6084/m9.figshare.hgv.2375
